# Resolvin D1 and D2 inhibit tumour growth and inflammation via modulating macrophage polarization

**DOI:** 10.1111/jcmm.15436

**Published:** 2020-05-29

**Authors:** Kai Shan, Ninghan Feng, Jing Cui, Shunhe Wang, Hongyan Qu, Guoling Fu, Jiaqi Li, Heyan Chen, Xiaoying Wang, Rong Wang, Yumin Qi, Zhennan Gu, Yong Q. Chen

**Affiliations:** ^1^ Wuxi School of Medicine Jiangnan University Wuxi China; ^2^ School of Food Science and Technology Jiangnan University Wuxi China; ^3^ Department of Urology Wuxi No. 2 People’s Hospital Wuxi China

**Keywords:** M1 macrophage, M2a macrophage, PKA, resolvin D, tumour‐associated macrophage

## Abstract

Plastic polarization of macrophage is involved in tumorigenesis. M1‐polarized macrophage mediates rapid inflammation, entity clearance and may also cause inflammation‐induced mutagenesis. M2‐polarized macrophage inhibits rapid inflammation but can promote tumour aggravation. ω‐3 long‐chain polyunsaturated fatty acid (PUFA)‐derived metabolites show a strong anti‐inflammatory effect because they can skew macrophage polarization from M1 to M2. However, their role in tumour promotive M2 macrophage is still unknown. Resolvin D1 and D2 (RvD1 and RvD2) are docosahexaenoic acid (DHA)‐derived docosanoids converted by 15‐lipoxygenase then 5‐lipoxygenase successively. We found that although dietary DHA can inhibit prostate cancer in vivo, neither DHA (10 μmol/L) nor RvD (100 nmol/L) can directly inhibit the proliferation of prostate cancer cells in vitro. Unexpectedly, in a cancer cell‐macrophage co‐culture system, both DHA and RvD significantly inhibited cancer cell proliferation. RvD1 and RvD2 inhibited tumour‐associated macrophage (TAM or M2d) polarization. Meanwhile, RvD1 and RvD2 also exhibited anti‐inflammatory effects by inhibiting LPS‐interferon (IFN)‐γ‐induced M1 polarization as well as promoting interleukin‐4 (IL‐4)‐mediated M2a polarization. These differential polarization processes were mediated, at least in part, by protein kinase A. These results suggest that regulation of macrophage polarization using RvDs may be a potential therapeutic approach in the management of prostate cancer.

## 1 INTRODUCTION

Macrophage possesses multiple functions in immunomodulation and tissue repair. Plastic polarization bestows the exact role of macrophage in diverse biological process. According to the Th1‐Th2 classification, macrophage polarization can be roughly divided into M1 and M2 types.^1^ Classically activated M1 macrophage can be found in tissues suffering acute inflammation or be experimentally induced by lipopolysaccharide (LPS) and interferon gamma (IFN‐γ). M1 macrophage plays a pro‐inflammatory role by increasing the concentrations of superoxide anions, oxygen radicals and nitrogen radicals as well as secreting inflammatory factors including interleukin 1β (IL‐1β), IL‐6 and tumour necrosis factor α (TNF‐α).^2^, ^3^ On the one hand, M1 macrophage can execute pathogen clearance and promote tumour cell killing via activating CD8^+^ T cell and NK cell^4^, ^5^, ^6^, ^7^, ^8^; on the other hand, M1 macrophage also causes inflammation and involves various pathological process such as insulin resistance and inflammation‐associated mutagenesis.^9^, ^10^ Activated M2 macrophage can be divided into various subsets including M2a, M2b, M2c and M2d (aka. tumour‐associated macrophage, TAM).^4^ Although these M2 subsets share some markers (eg CD206 and CD163) and immunosuppressive functions, different subsets are induced by different mechanisms and have diverse physiological functions.^11^, ^12^ M2a, induced by IL‐4 and/or IL‐13, is the mostly studied M2 subset. Immunosuppressive M2a macrophage expresses scavenger receptors and secretes IL‐10, transforming growth factor β (TGF‐β) and C‐C motif chemokine ligand 17 (CCL17).^4^, ^13^ The immunomodulatory functions of M2b and M2c are similar to those of M2a but have distinct inducers (immune complex for M2b and glucocorticoids for M2c).^4^, ^14^ In general, M2a, M2b and M2c tend to attenuate inflammation and are considered as physiologic inhibitors to M1 macrophage.^15^ Different from above‐mentioned three M2 subsets, TAM is a highly heterogeneous collection with diverse activation modes and markers among different tumour tissues. The exact inducer of TAM is still not clear and might include the combinations among vascular endothelial growth factors (VEGFs), TGF‐β, IL‐4, CCL2, colony‐stimulating factors (CSFs) and some extracellular matrix components. Therefore, common methods to acquire certain TAM are to isolate them from target tumour tissues in vivo or from the cancer cell‐macrophage co‐culture system in vitro. Despite the heterogeneity, different TAMs play a similar tumour promotive role in the microenvironment via secreting immunosuppressive factors (eg IL‐10), angiogenic factors (eg VEGFs) and growth factors (eg EGF).^7^, ^16^

Docosahexaenoic acid (DHA) which is a dietary ω‐3 polyunsaturated fatty acid (PUFA) exhibits remarkable anti‐inflammatory effect, and this effect largely attributes to its oxidation products including resolvin, maresin and protectin.^17^ Resolvin D (RvD) comprises a series of lipoxygenase metabolites from DHA and in which RvD1 and RvD2 get the most attentions. 15‐lipoxygenase (ALOX15) or aspirin‐modified cyclooxygenase‐2 (COX‐2) converts DHA to 17‐hydroxy DHA (17‐HDHA) and then 5‐lipoxygenase (ALOX5) metabolized 17‐HDHA to RvD1, RvD2, RvD3 and RvD4.^18^ In general, RvDs exhibit anti‐inflammatory effects via modulating the activation of monocytes, macrophages, T lymphocyte and epithelial cell.^19^, ^20^, ^21^ A switch of M1 to M2 polarization and consequent decrease of pro‐inflammatory mediators were reported as the critical mechanism of RvDs’ anti‐inflammatory function.^17^, ^19^, ^22^, ^23^ RvD1 can induce higher levels of reparative macrophages expressing typical M2‐like marker, CD206, in heart failure mice.^24^ RvDs inhibit murine abdominal aortic aneurysm formation and increase M2 macrophage polarization^17^, ^19^, ^22^, ^23^ and may also improve cognition in mild cognitive impairment patients.^19^, ^23^

As tumour environment tends to induce a M2‐like TAM, blocking TAM polarization could be a potential anti‐tumour therapeutic strategy.^25^ RvD was reported to suppress tumour growth by enhancing clearance of debris via macrophage phagocytosis^26^ and to reduce the number of cancer mediator‐induced CD11b^+^Ly6G^−^ myeloid cells.^27^ In addition, some studies have revealed the anti‐tumour effect of ω‐3 PUFAs is ALOX5‐ or ALOX15‐dependent.^28^, ^29^, ^30^ In light of these literature, we wonder whether RvD can affect TAM polarization. In this study, we found that RvDs had the opposite effect on M2a and TAM (M2d), namely RvD1 and RvD2 promoted M2a but inhibited TAM polarization. This process depended on RvD induced up‐regulation of PKA pathway.

## 2 MATERIALS AND METHODS

### 2.1 *Pten*‐knock out mice and diets

Prostate‐specific *Pten* knockout (*Pten* ^−/−^) mice were generated as described previously.^31^ All procedures were approved by the ethics committee of Jiangnan University. The prostate tissue was weighed, embedded into paraffin, cut into 5‐micron‐thick sections and stained with haematoxylin and eosin (H&E). Immunohistochemical staining was performed to detect the expression of total macrophages marker. The operation was according to previous studies of our laboratory.^31^, ^32^

Diet formulas were from the custom animal diet laboratory of the Animal Resources Program at Wake Forest University (US). According to our previous researches,^31^, ^32^ the ω‐6 diet was based on a typical American diet consisting of an ω‐6 to ω‐3 ratio of 40:1, 397 kcal/100 g with 30% of energy from fat, 50% from carbohydrates and 20% from proteins. And the isocaloric ω‐3 diet had an ω‐6 to ω‐3 ratio of 1:1.

### 2.2 Cell culture

Human prostate cancer cell line PC3 (CRL1435; ATCC), 22RV1 (CRL2505; ATCC), monocytic leukaemia cell line THP‐1 (TIB‐202; ATCC) and mouse fibroblast cell L929 (CCL1; ATCC) were provided by Shanghai Institute of Cell Biology, Chinese Academy of Sciences, Shanghai, China. PC3 and 22RV1 were maintained in RPMI 1640 supplemented with 5% (v/v) foetal bovine serum (FBS, 10099141; Gibco). THP‐1 was grown in RPMI 1640 medium supplemented with 5% (v/v) FBS, 200 μmol/L glutamine (Thermo Fisher Scientific) and 0.2 μmol/L β‐mercaptoethanol (Sigma). L929 cells were maintained in Dulbecco's modification of Eagle's medium (DMEM) supplemented with 5% (v/v) FBS.

Bone marrow‐derived macrophage (BMDM) was isolated from mouse tibia according to Spring Harbor Protocols.^33^ In brief, femur and tibia bones are collected from 6 to 8 weeks male C57BL6/J mice and bone marrow cells were flushed out using Hank's buffer. After lysis of red blood cells. BMDM cells are cultured in BMDM growth medium (70% DMEM complete medium, 30% L929 cell supernatant).

### 2.3 Macrophage polarization

Differentiation of THP‐1 cells into M0, M1 or M2a macrophages was performed as previously described.^34^ Briefly, THP‐1 cells were differentiated into M0 macrophages by incubation with 100 nmol/L phorbol 12‐myristate 13‐acetate (PMA, P8139; Sigma) for 24 hours. Then, the cells were transferred to PMA‐free media for another 24 hours to obtain resting macrophages (M0). These cells were then polarized to M1 macrophages by treating with 100 ng/mL LPS (L4516, Sigma) and 20 ng/mL hIFN‐γ (285‐IF; R&D systems) for 72 hours or to M2a macrophages by treating with 20 ng/mL hIL‐4 (204‐IL; R&D systems) for 72 hours. For BMDM polarization, cells were similarly stimulated with LPS/mIFN‐γ or mIL‐4. To obtain TAM, M0 THP‐1 cells were incubated with supernatant of cancer cells or co‐cultured with cancer cells for 3 days. During TAM polarization, media with or without RvD were renewed at day 2 and collected at day 3 as conditioned medium (CM) or RvDCM, respectively. RvD1 (872993‐05‐0) and RvD2 (810668‐37‐2) were purchased from Cayman chemical. For co‐culture assay, transwell inserts with 0.4 μm pore size were chosen to avoid cell shuttle.

### 2.4 Western blot

Proteins were separated by SDS‐PAGE, transferred onto an NC membrane (45‐004‐001, GE Healthcare) and then probed with relevant antibodies (Table S2) at 4°C overnight. HRP‐labelled goat anti‐mouse or anti‐rabbit IgG secondary antibodies were used for ECL detection (WBKLS0500; Sigma).

### 2.5 RNA extraction and quantitative real‐time PCR (qPCR)

Total RNA was isolated using TRIZOL reagent (15596018, Thermo Fisher Scientific) according to the manufacturer's protocol. Then, reverse transcription was performed using Prime Script^®^ RT reagent Kit with gDNA Eraser (PR047A; Takara). Real‐time PCR was performed on CFX96 Real‐Time System (BIO‐RAD) using SYBR Green PCR master mix (4367659; Thermo Fisher Scientific). Primers used for qPCR were listed in Table S1. All experiments were performed in triplicate, and the mRNA level of GAPDH was chosen as the internal reference. Fold changes of target genes were calculated using the 2^(‐ΔΔCT)^.

### 2.6 Flow cytometry analysis

Cells were detached with trypsin and re‐suspended in DPBS as single cell suspensions. Then, the suspensions were incubated with human or mouse FcR Blocking Reagent (Miltenyi Biotec) for 30 minutes at 4°C. Surface antigens were then stained directly, while intracellular antigens were stained after membrane permeabilization. Buffers and antibodies used for flow cytometry analysis were listed in Table S2. Isotype‐matched controls were included. Stained cells were analysed on an Attune NxT flow cytometer (Thermo Fisher Scientific).

### 2.7 MTT assay

To determine cell proliferation, MTT (Thiazolyl Blue Tetrazolium Bromide) assay was performed. In brief, medium was replaced by 0.5 mg/mL MTT (M5655; Sigma) in serum‐free medium. MTT solution was discarded after 4 hours incubation under normal culture conditions, and insoluble formazan was dissolved by DMSO. Values of OD570 and OD630 were recorded to evaluate cell count.

### 2.8 ELISA

Sandwich ELISA was performed to detect cytokines in TAM supernatant. The supernatant was directly used for ELISA in a 96‐well plate without dilution or freeze‐thaw. All operations were performed in accordance with manufacturer's protocol. After adding stop solution, plates were read immediately using a microplate reader set to 450 and 540 nm (reference wavelength). The concentration of cytokines was calculated according to the standard curve. Three independent experiments were performed. The information of kits can be found in Table S2.

### 2.9 Statistics

All data were analysed using GraphPad Prism 6 software. The results from animal experiments were shown as mean ± SEM, and the results from cultured cell were shown as mean ± SD (n ≥ 3). Two‐tailed Student's *t* test was used for the statistical comparison of two groups. One‐way analysis of variance (ANOVA); then, Tukey's test was used for multiple comparisons. *P* < .05 was considered significant. When using letters to show statistical differences, there was NO significant difference between groups marked with the same letter and there were significant differences between any two groups marked with different letters.

## 3 RESULTS

### 3.1 RvD1 and RvD2 reduce the ability of macrophage to stimulate cancer cell proliferation

**FIGURE 1:**
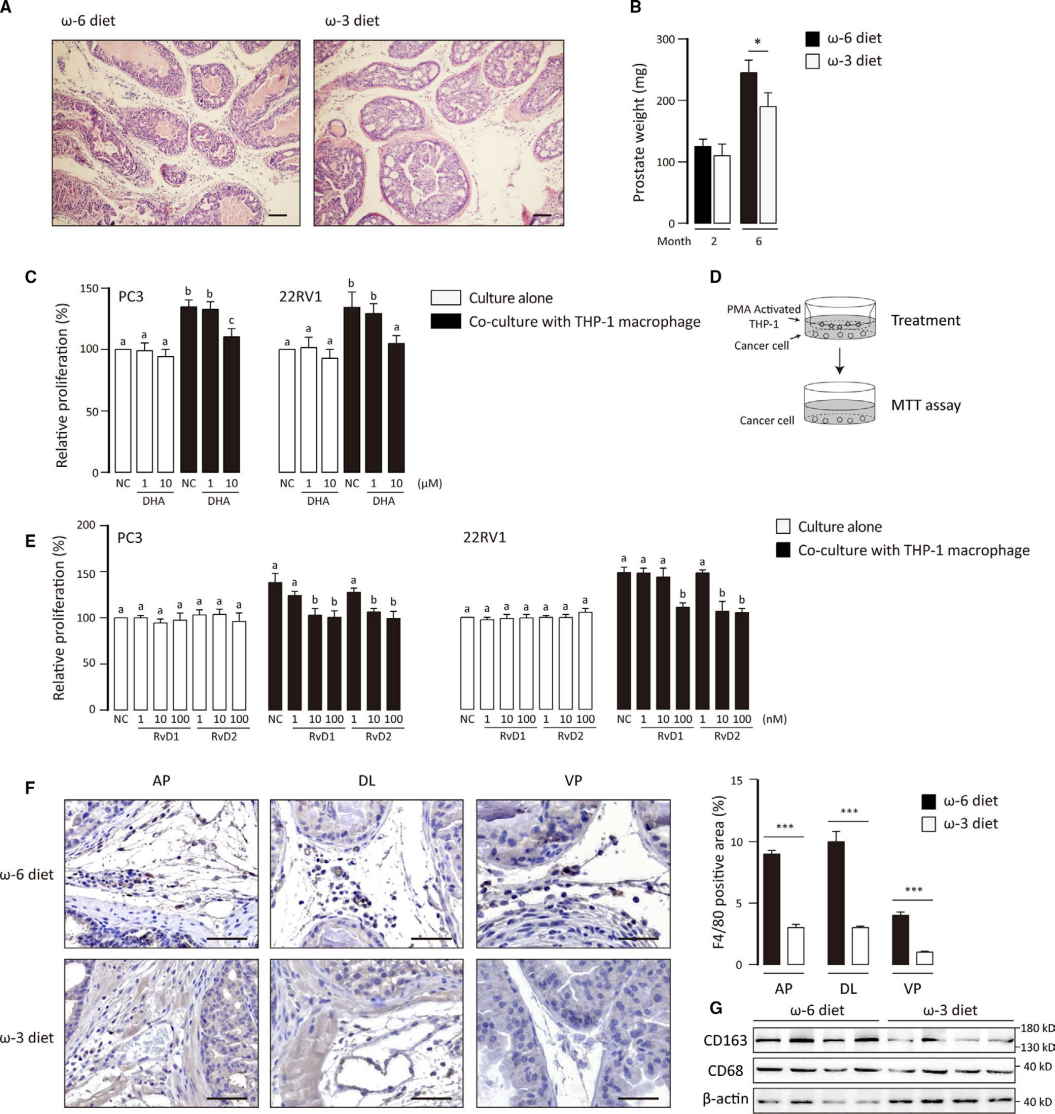
Effects of DHA and RvD on prostate cancer proliferation. A, H&E staining of prostate tissues from six‐month‐old *Pten* ^−/−^ mice. Scale bar: 100 μm. B, Prostate weights of two‐ and six‐month‐old *Pten* ^−/−^ mice. Data were shown as mean ± SEM (n = 5). Student's *t* test was performed. *, *P* < .05. C and E, PC3 and 22RV1 cell were cultured alone or co‐cultured with M0 THP‐1 cell and received treatments with fatty acid and RvDs for 48 h. Cell proliferation was determined by MTT assays. Results are expressed as percentages relative to the control and correspond to the means ± SD of three independent experiments. For experiments where necessary, ANOVA (Tukey's test) was performed, and *P* < .05 was considered significant. Statistical differences were found among groups marked with different letters. D, Diagram of the co‐culture system. The pore size of inserts was 0.4 μm. F, Immunohistochemical staining of F4/80 was performed to determine macrophage infiltration. AP, DL and VP: anterior, dorsolateral, and ventral prostate. Scale bar: 100 μm. F4/80 positive area (%) was calculated with ImageJ software. Data were shown as mean ± SEM (n = 50) ***P < .001. G, Detect expression of total macrophages and M2‐like TAM markers in *Pten* ^−/−^ prostate with western blot

Prostate‐specific knockout of *Pten* (*Pten* ^−/−^) induces prostatic intraepithelial neoplasia (PIN) and prostate cancer in mice.^35^ To verify the effect of dietary DHA on mouse prostate cancer, we generated two diets with high ω‐6 PUFAs (mainly provided by Safflower seed oil) and high ω‐3 PUFAs (mainly provided by fish oil with 70% DHA). Compared with ω‐6 PUFA diet which induced significant extension of malignant cells, ω‐3 PUFA diet attenuated the pathogenesis of prostate cancer in *Pten* ^−/−^ mice (Figure 1A). And, the weight of the prostate was also reduced in mice with ω‐3 PUFA diet (Figure 1B). Our previous researches also indicated that ω‐3 PUFA diet reduced Ki67 expression and caspase 3 cleavage in the prostate of *Pten* ^−/−^ mice.^31^, ^32^ However, as the active component, direct addition of DHA into culture could not inhibit cancer cell proliferation under our experimental conditions (Figure 1C). Thus, we reasoned that the metabolites of ω‐3 PUFA and the involvement of tumour microenvironment may mediate ω‐3 PUFA induced tumour inhibition.

ALOX5 and ALOX15 which convert DHA to resolvin Ds (RvDs) were highly expressed in prostate cancer.^36^ And they were also expressed in prostate cancer from mice fed with both ω‐6 and ω‐3 PUFA diet (Figure S1A). RvDs showed their anti‐tumour effects against some cancer cells.^27^, ^37^, ^38^ Unexpectedly, neither RvD1 nor RvD2 inhibited proliferation of prostate cancer cells directly (Figure 1E).

Macrophage which has a M2‐like phenomenon is an important tumour promotive component in tumour microenvironment. We found that ω‐3 PUFA diet strongly decreased the infiltration of total macrophage (Figure 1F). Moreover, referred to CD68 (a total macrophage marker), CD163 expression (an M2‐like macrophage marker) was also inhibited by ω‐3 PUFA diet (Figure 1G). These suggested that tumour‐associated macrophage (TAM) may involve with *Pten* ^−/−^ tumour development. As expected, proliferation of cancer cells was significantly promoted by the co‐cultured THP‐1 macrophage. Interestingly, DHA and RvDs effectively reverse this process although they cannot directly inhibit tumour growth (Figure 1C‐E). These results suggest that macrophage co‐cultured with cancer cell can promote the proliferation of cancer cells in which RvD1 and RvD2 can dampen the oncogenic crosstalk between cancer cells and macrophages.

### 3.2 RvD1 and RvD2 suppress TAM polarization

**FIGURE 2:**
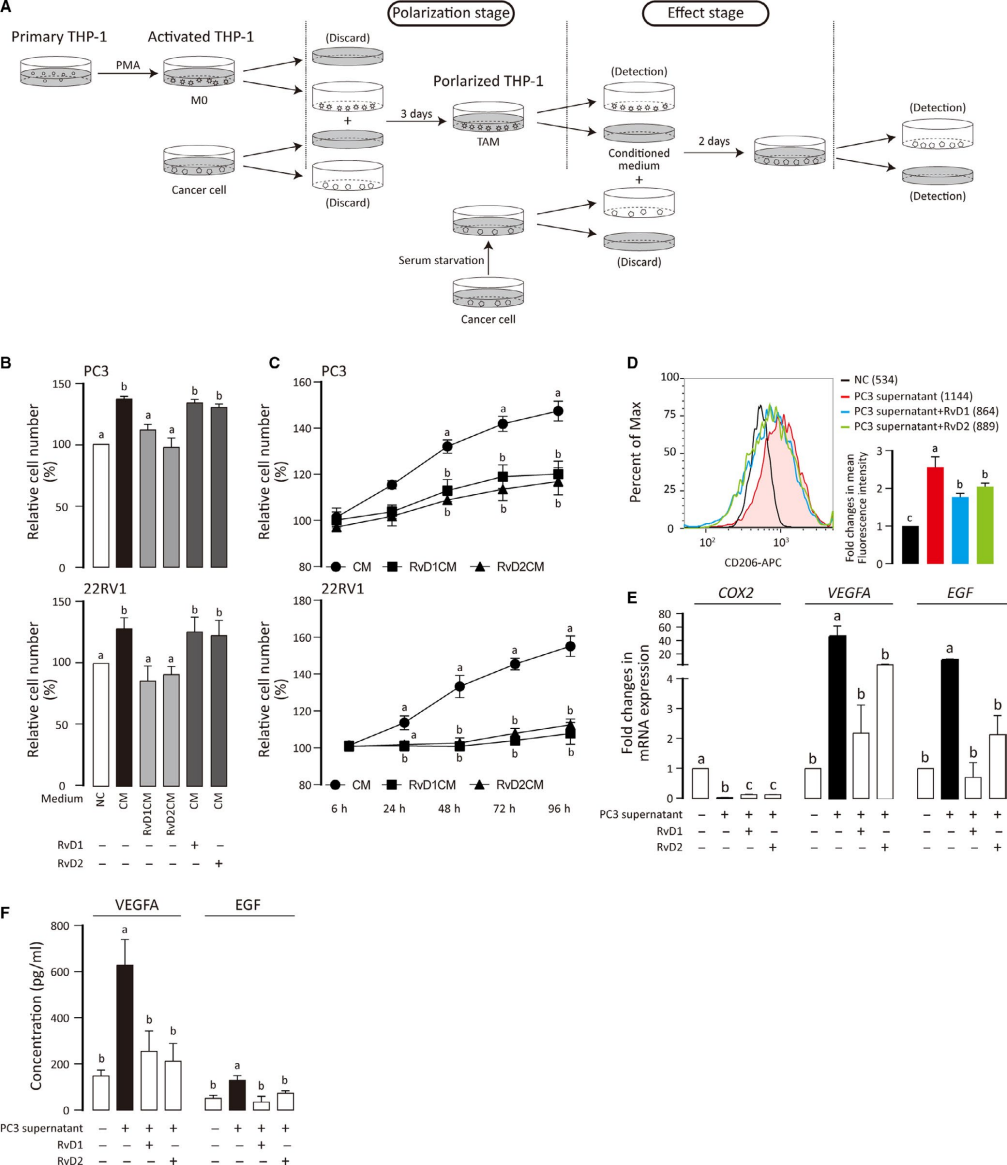
Suppression of TAM polarization by RvD1 and RvD2. A, Flow chart shows THP‐1 priming, TAM polarization and effect stages. B, PC3 and 22RV1 were treated with TAM‐conditioned medium (CM), RvD treated CM (RvDCM) and RvD plus CM for 48 h. The results are expressed as percentages relative to the control and correspond to the means ± SD of three independent experiments. C, Cell growth curves were displayed according to the results of MTT assay. PC3 and 22RV1 were treated with CM, respectively. Four groups of cells (NC, CM, RvD1CM and RvD2CM) were used for MTT assay at different time points. D, Primed THP‐1 cells were treated, stained for CD206 and analysed by flow cytometry. Mean fluorescence intensities were shown. Three independent experiments were integrated into a bar chart. ANOVA (Tukey's test) was performed and *P* < .05 was considered as significant. Statistical differences were found among groups marked with different letters. E, Messenger RNA level of indicated genes was measured by qPCR. Results are shown as means ± SD of three independent experiments. ANOVA (Tukey's test) was performed, and *P* < .05 was considered as significant. Statistical differences were found among groups marked with different letters. F, ELISA was performed to determine the concentration of VEGFA and EGF in TAM supernatant. Results are shown as means ± SD of three independent experiments. ANOVA (Tukey's test) was performed and *P* < .05 was considered as significant. Statistical differences were found among groups marked with different letters

Cancer cells generate a tumour‐promoting microenvironment in which TAM is both the ‘victim’ (polarization stage) and the ‘perpetrator’ (effect stage).^39^ In order to determine the mechanism of RvDs’ effect, we separated these two stages. Primed THP‐1 was treated by supernatant of cancer cells (polarization stage), and then, macrophage‐conditioned medium was transferred to newly seeded cancer cells (effect stage) (Figure 2A). Consistent with the co‐culture results, TAM‐conditioned medium (CM) promoted the growth of cancer cells and RvD also diminished the stimulatory activity of CM. Of note, only adding RvD1 and RvD2 in the polarization stage but not the effect stage inhibits the proliferation of cancer cells (Figure 2B). At the same time, cell growth curve showed that CM accelerated the growth of tumour cells, while RvDCM could alleviate this effect (Figure 2C). Thus, RvD1 and RvD2 affected TAM polarization rather than TAM’s effect.

TAM expresses some of M2 macrophage markers including CD206.^40^ Meanwhile, TAM expresses highly VEGFA and epidermal growth factor (EGF) and promotes angiogenesis and carcinogenesis.^41^, ^42^ We found that the supernatant of PC3 prostate cancer cells promoted the expression of CD206, VEGFA and EGF in THP‐1 indicating the polarization of TAM. However, RvD1 and RvD2 reversed the changes of CD206, VEGFA and EGF expression (Figure 2D‐F). Interestingly, we also found that *COX‐2*, a hallmark of M1 macrophage, was down‐regulated in TAM and could also be partially reversed by RvD (Figure 2E). We also noted that the supernatant of M0 THP‐1 had no effect on the growth of tumour cells, and the effect of RvDs on M0 THP‐1 was trifling (Figure S1B‐D). These results suggest that RvD1 and RvD2 can inhibit TAM polarization, and this provides a potential mechanism for their anti‐tumour effect.

### 3.3 RvD1 and RvD2 promote M2a polarization

**FIGURE 3:**
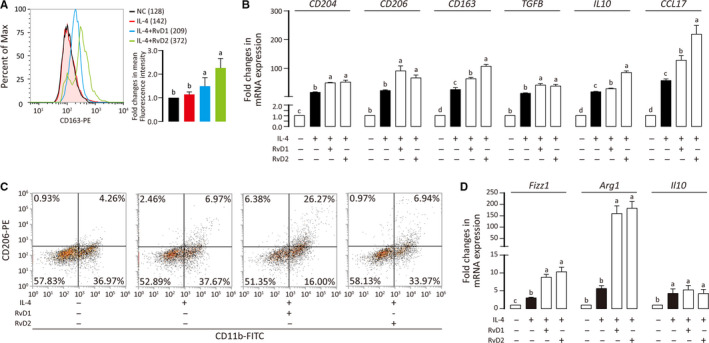
Promotive effect of RvD1 and RvD2 on M2a polarization. A, Primed THP‐1 was treated with IL‐4 and RvDs, stained for CD163, and analysed by flow cytometry. Mean fluorescence intensities were shown. Three independent experiments were integrated into a bar chart. ANOVA (Tukey's test) was performed and *P* < .05 was considered as significant. Statistical differences were found among groups marked with different letters. B, Messenger RNA level of indicated genes were quantified by qPCR and results are shown as means ± SD of three independent experiments. C, BMDM cells were treated with IL‐4 and RvDs, stained for CD11b and CD206, and analysed by flow cytometry. D, Messenger RNA level of indicated genes were determined with and results are shown as means ± SD of three independent experiments. For experiments where necessary, ANOVA (Tukey's test) was performed and *P* < .05 was considered as significant. Statistical differences were found among groups marked with different letters

TAM aka. M2d macrophage is a subset of M2 macrophage.^43^ Thus, it is necessary to explore if resolvins’ inhibitory effect on TAM polarization could affect other anti‐inflammatory subsets of M2 macrophage, for example M2a. IL‐4 can induce a classical M2a polarization with an increased expression of CD204, CD163, TGF‐β, IL‐10, CCL17 and CD206.^44^, ^45^, ^46^ Contrary to TAM, RvD1 and RvD2 up‐regulated the expression of these M2a markers and factors (Figure 3A,B). Similar results were observed not only in THP‐1 macrophage but also in BMDM. RvD1 and RvD2 increased the markers of M2a in BMDM including *Fizz1* and *Arg1* (Figure 3C,D). These results indicate that RvD1 and RvD2 preferentially inhibit polarization of TAM (M2d) but promote polarization of M2a macrophage.

### 3.4 RvD1 and RvD2 inhibit M1 polarization

**FIGURE 4:**
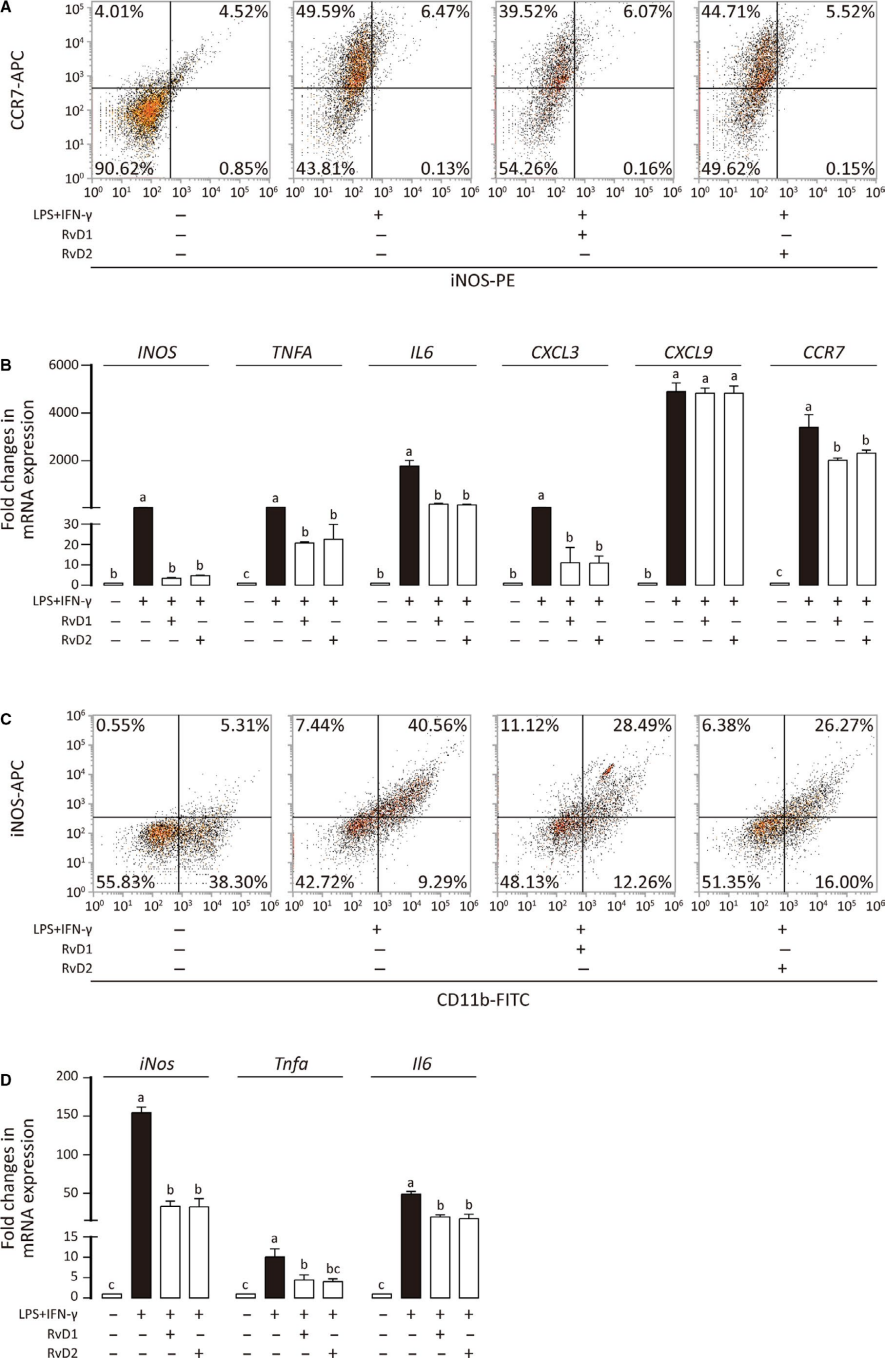
Inhibitory effect of RvD1 and RvD2 on M1 polarization. A, Primed THP‐1 was treated with LPS + IFN‐γ in the presence or absence of RvDs, stained for CCR7 and iNOS, and analysed by flow cytometry. B, Messenger RNA level of indicated genes was measured by qPCR, and results are shown as means ± SD of three independent experiments. C, BMDM was treated with LPS + IFN‐γ in the presence or absence of RvDs, stained for CD11b and iNOS, and analysed by flow cytometry. D, Messenger RNA level of indicated genes was measured by qPCR, and results are shown as means ± SD of three independent experiments. For experiments where necessary, ANOVA (Tukey's test) was performed and *P* < .05 was considered as significant. Statistical differences were found among groups marked with different letters

We also evaluated the role of RvD1 and RvD2 on M1 macrophage polarization. It is well documented that LPS and IFN‐γ induce THP‐1 cells to M1 macrophage with up‐regulation of C‐C chemokine receptor type 7 (CCR7), inducible nitric oxide synthases (iNOS), TNF‐α, C‐X‐C motif chemokine ligand 3 (CXCL3), IL‐6 and CXCL9.^47^, ^48^, ^49^ RvD1 and RvD2 treatments reduced all these markers except *CXCL9* (Figure 4A,B). Similarly, the M1 polarization of BMDM was also inhibited by RvD1 and RvD2 (Figure 4C,D). These results indicate that RvD1 and RvD2 can significantly inhibit the polarization of M1 macrophages supporting their anti‐inflammatory role.

### 3.5 RvD1 and RvD2 modulate macrophage polarization via PKA pathway

**FIGURE 5:**
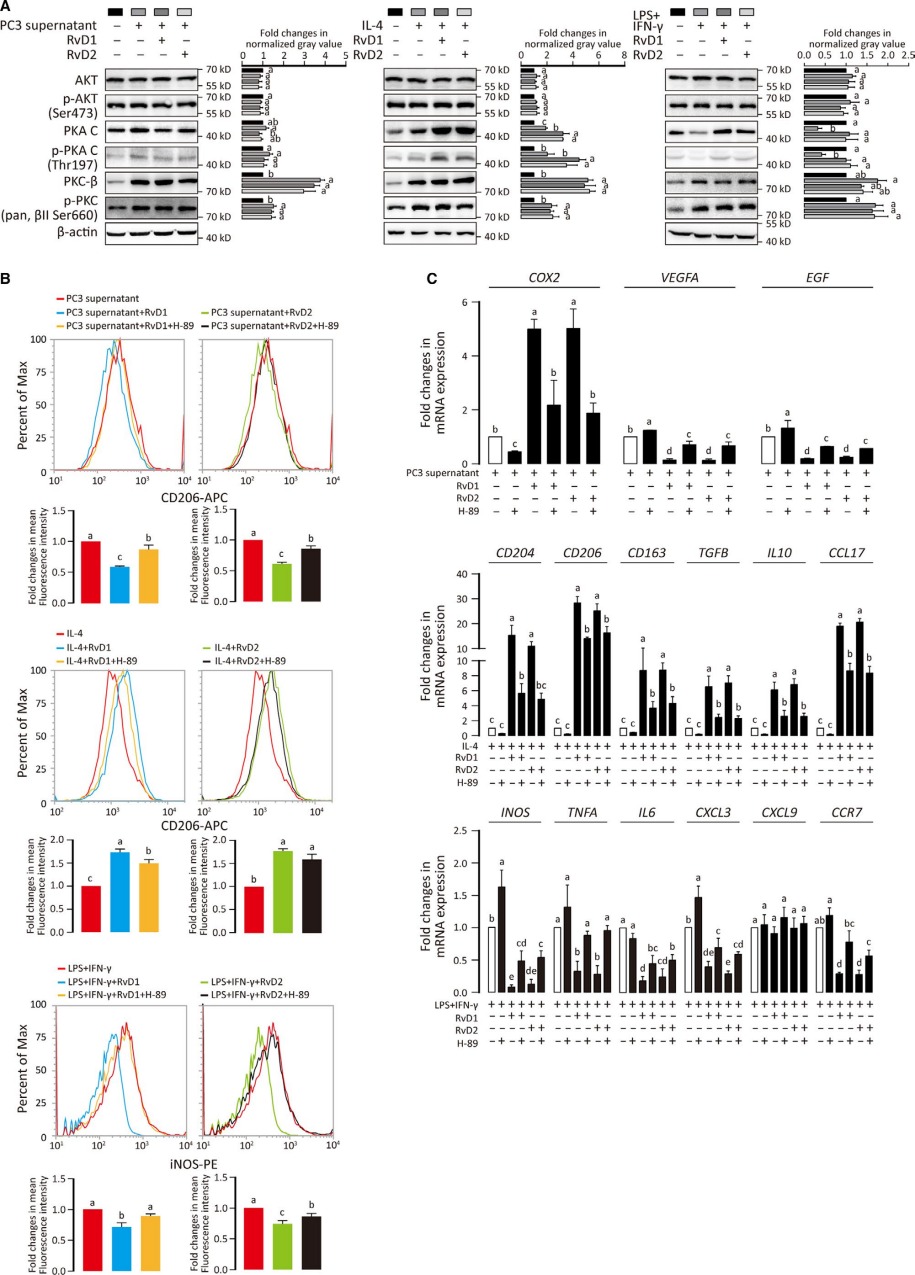
RvD1 and RvD2 modulate macrophage polarization via PKA pathway. A, Western blot was performed to check the expression and phosphorylation of AKT1, PKA‐C and PKC‐β in TAM, M2a and M1 macrophages. The grey values of each band were extracted by ImageJ software. Results were shown as fold changes (mean ± SD), and ANOVA was performed. *P* < .05 was considered as significant. Statistical differences were found among groups marked with different letters. B, PKA inhibitor H‐89 was added to examine the role of PKA in polarization of TAM, M2a and M1 macrophages. Cells were stained for CD206 and iNOS then analysed by flow cytometry. Mean fluorescence intensities from three independent experiments were integrated into bar charts. ANOVA (Tukey's test) was performed, and *P* < .05 was considered as significant. Statistical differences were found among groups marked with different letters. C, Messenger RNA level of indicated genes was measured by qPCR, and results are shown as means ± SD of three independent experiments. ANOVA (Tukey's test) was performed, and *P* < .05 was considered as significant. Statistical differences were found among groups marked with different letters

The results above raised a question how RvDs regulate different macrophage subsets at the same time. There are three known RvD receptors, GPR32 and ALX/FPR2 for RvD1 and GPR18 for RvD2.^50^, ^51^ These receptors are expressed on macrophage (Figure S1E,F) and regulate cell function via cAMP/PKA pathway (ALX/FPR2, GPR32 and GPR18),^52^, ^53^, ^54^ PI3K/AKT pathway (ALX/FPR2 and GPR18)^55^, ^56^, ^57^ and PKC pathway (ALX/FPR2 and GPR32).^58^, ^59^ Therefore, we explored the classical activation of PKA, AKT and PKC pathways during RvD involved macrophage polarization. Total and phosphorylated PKA‐C (Thr197) were reduced in M1 while up‐regulated in M2a and TAM macrophages (Figure 5A). AKT had no significant change among M0, M1, M2a and TAM macrophages while both total and phosphorylated PKCβ were increased in M1, M2a and TAM macrophages (Figure 5A). Up‐regulated PKA and p‐PKA might be inhibitory to M1 but promotive to M2 polarization. In TAM polarization, however, effect of RvD on PKA was less pronounced (Figure 5A). Therefore, it seems that the differential effect of RvDs on various subsets of macrophages is via its regulation of PKA pathway. In view of the potential role of the PKA pathway, we explored the effect of PKA inhibition on macrophage polarization. Upon H‐89 (a PKA inhibitor) treatment, the effects of RvDs on M1, M2a and TAM polarization were all reversed except RvD2 regulated M2a (Figure 5B,C). These data suggest that PKA pathway plays an important role in macrophage polarization. The regulation of RvD on macrophage polarization partly depends on PKA pathway.

## 4 DICUSSION

Previous studies have reported that RvDs induce a switch of M1 polarization to M2 polarization,^17^, ^19^, ^22^, ^23^ while their role on TAM polarization is still unclear. Our study found that RvD1 and RvD2 can regulate the polarization of M1, M2a and prostate cancer‐associated TAM. Besides the co‐culture system, we adopted a medium transfer model and identified that RvD1 and D2 affected macrophage directly rather than tumour cells. This finding suggests that RvD1 and D2 can exert their anti‐tumour effect on multiple stages during tumorigenesis. The inhibition of M1 and promotion of M2a macrophage attenuate the mutagenic inflammation before tumour initiation and the modulation of TAM reduces the support for tumour cell proliferation.

It is generally believed that the signal pathway activated by RvDs originate from their receptors. RvD1 interacts with both GPR32 and ALX/FPR2 while RvD2 binds to GPR18,^50^, ^51^ even though, ligands of these receptors are not limited to resolvins. However, signalling pathway activated by RvD may be different from those activated by other ligands. For example, GPR32 and GPR18 are both considered as the Gi type G protein‐coupled receptor,^53^, ^54^ but RvD1 and RvD2 have been repeatedly proven to up‐regulate cAMP.^53^, ^60^ Here, we also found that RvD1 and RvD2 could increase both phosphorylated and total PKA in M1 and M2a macrophages. This suggests that RvDs may activate their receptors through recruiting Gs type rather than generally reported Gi type effectors.

Cyclic AMP‐PKA pathway is a critical determinant in M1‐M2a polarization, potentially regulating the direction of macrophage polarization.^61^ In this study, we showed that both total PKA and phosphorylated PKA were down‐regulated in M1 macrophage whereas up‐regulated in M2a and TAM. IFN‐γ‐signal transducer and activator of transcription 1 (STAT1) pathway determines M1 polarization and IL‐4‐STAT6 pathway leads to M2a polarization.^62^ PKA regulatory IIα subunit (PRKAR2A) can bind to the transmembrane domain of IFN‐γ receptor and suppressed Janus kinase 2 (Jak2)‐STAT1 pathway. In contrast, PKA activates cAMP response element‐binding protein (CREB) and up‐regulates the transcription of M2 polarization elicited by IL‐4.^63^, ^64^ RvD restored reduced PKA in M1 and amplified enhanced PKA in M2a. This provides a potential mechanism to explain why RvD can inhibit M1 and promote M2a polarization at the same time.

Inducers of TAM may include cytokines (IL4, IL‐13 and IL‐10), lipid mediators (PGE_2_), chemokines (CCL2 and CCL22), growth factors (CSF1, VEGFs and EGF), immune complex and various extracellular molecules (ROS, L‐arginine, PD‐1, ANG2, HMGB1 and even low oxygen).^65^ TAM also has some molecular characteristics of both M1 and M2 including low NF‐κB activation, high hypoxia‐inducible factor (HIF)‐1α, high iNOS and high Arg‐1.^62^, ^66^ It is unclear though what factor(s) from prostate cancer cells initiates TAM polarization and how activation (activation degree, activation site and even subcellular localization) of PKA promotes the polarization process. However, our study observed a slight but stable increase of PKA in TAM and we also found that PKA inhibitor, H‐89, can reduce TAM polarization. These suggest that activation of PKA pathway is necessary, but not sufficient for TAM polarization and RvD’s effect can be multi‐targeted. The activity of PKA depends on not only its activation but also its subcellular localization. A‐kinase anchoring proteins (AKAPs) is the controller of PKA localization. Microtubule‐binding protein MAP2 which is an AKAP can be significantly up‐regulated by ω‐3 PUFA rather than ω‐6 PUFA.^67^, ^68^ This provides another potential mechanism by which RvDs regulate MAP2 (or other AKAPs) and alter the subcellular localization of PKA so that influence the signal transduction downstream of PKA without affecting the activation of PKA itself.

## CONFLICT OF INTEREST

The authors declared that they have no conflicts of interest to this work.

## Supporting information

Fig S1

Table S1‐S2

## Data Availability

The data used to support this study are available from the corresponding author upon request.
