# Supplementary figures and images for: Resolvin D1 and D2 inhibit tumour growth and inflammation via modulating macrophage polarization
Source: J Cell Mol Med. 2020 May 29;24(14):8045–56. doi: 10.1111/jcmm.15436 (PMC7348143; doi:10.1111/jcmm.15436)

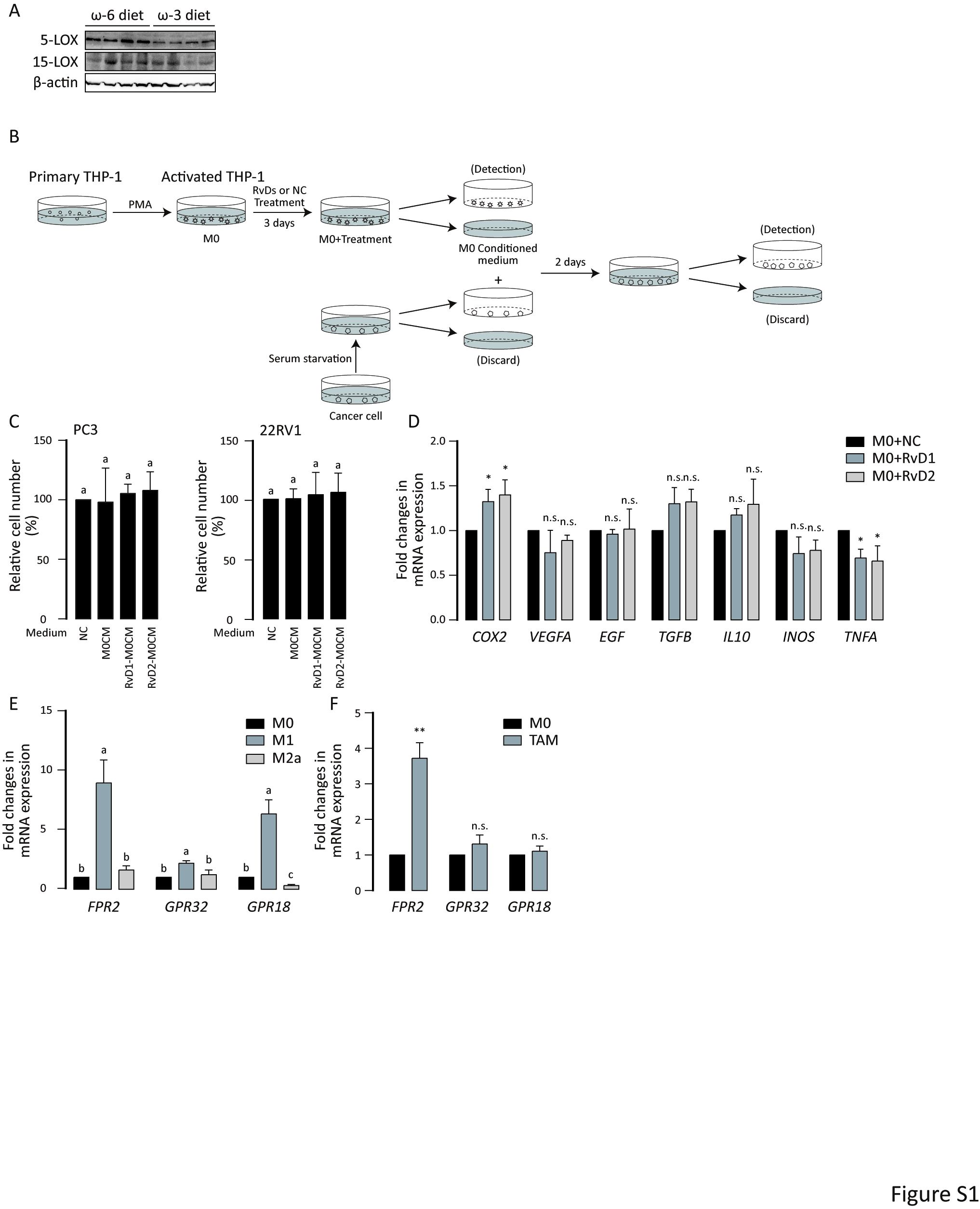

Supplement: Supplementary file 1 — Fig S1 [file JCMM-24-8045-s001.tif]
