# Supplementary material for: Resolvin D1 and D2 inhibit tumour growth and inflammation via modulating macrophage polarization
Source: J Cell Mol Med. 2020 May 29;24(14):8045–56. doi: 10.1111/jcmm.15436 (PMC7348143; doi:10.1111/jcmm.15436)
Supplement: Supplementary file 2 — Table S1‐S2 [file JCMM-24-8045-s002.docx]

**Table S1. Primers used in this study**

| Gene | Sequence (5'-3') F | Sequence (5'-3') R |
| --- | --- | --- |
| h*COX2* | TAAGTGCGATTGTACCCGGAC | TTTGTAGCCATAGTCAGCATTGT |
| h*EGF* | GGGAGCCTGAGCAGAAACTT | CTACAGGGCACGTGCAGTAA |
| h*VEGF* | CACCATGCAGATTATGCGGA | GAGGCTCCAGGGCATTAGAC |
| h*GAPDH* | GCCAGTAGAGGCAGGGATGATGTTC | CCATGTTCGTCATGGGTGTGAACCA |
| h*CD204* | TCCACTGAGAGGGATGAGAACT | TTCACTATCACAGGAGGACAC |
| h*CD206* | CTACTGTTATGTCGCTGGCAAA | GGATGGAAGCAAAGTGGATTAG |
| h*CD163* | AGCCATTATTACACACGTTCC | TTTTGTCACCAGTTCTCTTGGA |
| h*TGFB* | GAACCCGTTGATGTCCACTT | CACGTGGAGCTGTACCAGAA |
| h*IL10* | GTGGGTGCAGCTGTTCTCAGACT | AAAAGAAGGCATGCACAGCTCAG |
| h*CCL17* | CCCTGCACAGTTACAAAAACGA | GAGCCATTCCCCTTAGAAAGCT |
| h*INOS* | CACGGCCTTGCTCTTGTTTT | GTGATGCCCCAAGCTGAGA |
| h*TNFA* | GGCCAGAGGGCTGATTAGAGA | CTTCTGCCTGCTGCACTTTG |
| h*IL6* | TCTGAGGTGCCCATGCTACATTT | GCTGCAGGACATGACAACTCATC |
| h*CXCL3* | GTGGCTATGACTTCGGTTTGG | TGCCAGTGCTTGCAGACACT |
| h*CXCL9* | GTCCCTTGGTTGGTGCT | CATCTTGCTGGTTCTGATTGGA |
| h*CCR7* | GTAATCGTCCGTGACCTCATCTT | GCTGGTGGTGGCTCTCCTT |
| miNos | CTGATGGCAGACTACAAAGACG | TGGCGGAGAGCATTTTTGAC |
| m*Tnfa* | GCTACGACGTGGGCTACAG | CCCTCACACTCAGATCATCTTCT |
| m*Il6* | TTGGTCCTTAGCCACTCCTCC | TAGTCCTTCCTACCCCAATTTCC |
| m*Fizz1* | GGTCCCAGTGCATATGGATGAGACC | CACCTCTTCACTCGAGGGACAGTTG |
| m*Arg1* | TGGCTTGCGAGACGTAGAC | GCTCAGGTGAATCGGCCTTTT |
| m*Il10* | CGGTTAGCAGTATGTTGTCCAGC | CGGGAAGACAATAACTGCACCC |
| m*Gapdh* | TGTAGACCATGTAGTTGAGGTCA | AGGTCGGTGTGAACGGATTTG |
| h*FPR2* | AGTCTGCTGGCTACACTGTTC | TGGTAATGTGGCCGTGAAAGA |
| h*GPR32* | GTGATCGCTCTTGTTCCAGGA | GGACGCAGACAGGATAACCAC |
| h*GPR18* | CGCCACCTGCCTCAAGATTT | TGACCAAGTAGCACCCAATCAT |

**Table S2.** **Reagents and antibodies used in this study**

| Name | Vendor | Catalog number | Solvent | Final conc. |
| --- | --- | --- | --- | --- |
| Fetal bovine serum | Gibco, MA, US | 10099141 |  | 5% |
| L-Glutamine | Thermo Fisher Scientific, MA, US | 25030081 |  | 200 μM |
| Phorbol 12-myristate 13-acetate (PMA) | Sigma, MO, US | P8139 | DMSO | 100 nM |
| LPS | Sigma, MO, US | L4516 | DPBS | 100 ng/ml |
| hIFN-γ | R&D systems, Shanghai, China | 285-IF | DPBS | 20 ng/ml |
| hIL-4 | R&D systems, Shanghai, China | 204-IL | DPBS | 20 ng/ml |
| RvD1 | Cayman chemical, Shanghai, China | 872993-05-0 | Ethanol | 100 nM |
| RvD2 | Cayman chemical, Shanghai, China | 810668-37-2 | Ethanol | 100 nM |
| mIFN-γ | R&D systems, Shanghai, China | 485-MI | DPBS | 20 ng/ml |
| mIL-4 | R&D systems, Shanghai, China | 404-ML | DPBS | 20 ng/ml |
| TRIZOL | Thermo Fisher Scientific, MA, US | 15596018 |  |  |
| MTT | Sigma, MO, US | M5655 | DPBS | 0.5 mg/ml |
| H-89 | MedChem Express, Shanghai, China | HY-15979 | DMSO | 500 nM |
| FcR Blocking Reagent (mouse) | Miltenyi Biotec, Shanghai, China | 130-092-575 |  |  |
| FcR Blocking Reagent (human) | Miltenyi Biotec, Shanghai, China | 130-059-901 |  |  |
| PI/RNase staining buffer | BD Biosciences, Shanghai, China | 550825 |  |  |
| anti-hCD206-APC | Biolegend, CA, US | 321110 |  |  |
| anti-hCD163-PE | Invitrogen, MA, US | A15792 |  |  |
| anti-hiNOS-PE | Novus, CO, US | NBP2-22119 |  |  |
| anti-hCCR7-APC | eBioscience, MA, US | 17-1979-42 |  |  |
| anti-hCCR7-FITC | BD Biosciences, Shanghai, China | 561675 |  |  |
| anti-mCD206-PE | Biolegend, CA, US | 141705 |  |  |
| anti-mCD11b-FITC | eBioscience, MA, US | 11-0112-81 |  |  |
| anti-miNOS-APC | eBioscience, MA, US | 17-5920-80 |  |  |
| anti-AKT | Cell Signaling Technology, Shanghai, China | 9272 |  |  |
| anti-pAKT (Ser473) | Cell Signaling Technology, Shanghai, China | 9271 |  |  |
| anti-PKA Cα/β | R&D systems, Shanghai, China | MAB5908 |  |  |
| anti-PKA C (Thr197) | Cell Signaling Technology, Shanghai, China | 4781S |  |  |
| anti-PKC β | Proteintech, Hubei, China | 12919-1-AP |  |  |
| anti-pPKC (pan, βII Ser660) | Cell Signaling Technology, Shanghai, China | 9371S |  |  |
| anti-β actin | Sigma, MO, US | A1978 |  |  |
| anti-5 Lipoxygenase | Abcam, Shanghai, China | ab169755 |  |  |
| anti-15 Lipoxygenase 1 | Abcam, Shanghai, China | ab244205 |  |  |
| F4/80 | Abcam, Shanghai, China | ab111101 |  |  |
| CD163 | Abcam, Shanghai, China | ab182422 |  |  |
| CD68 | Abcam, Shanghai, China | ab125047 |  |  |
| Human VEGF DuoSet ELISA | Bio-Techne, Shanghai, China | DY293B |  |  |
| Human EGF DuoSet ELISA | Bio-Techne, Shanghai, China | DY236 |  |  |
